# Gonomics: uniting high performance and readability for genomics with Go

**DOI:** 10.1093/bioinformatics/btad516

**Published:** 2023-08-25

**Authors:** Eric H Au, Christiana Fauci, Yanting Luo, Riley J Mangan, Daniel A Snellings, Chelsea R Shoben, Seth Weaver, Shae K Simpson, Craig B Lowe

**Affiliations:** Department of Molecular Genetics and Microbiology, Duke University School of Medicine, Durham, NC 27710, United States; Department of Molecular Genetics and Microbiology, Duke University School of Medicine, Durham, NC 27710, United States; University Program in Genetics and Genomics, Duke University School of Medicine, Durham, NC 27710, United States; Department of Molecular Genetics and Microbiology, Duke University School of Medicine, Durham, NC 27710, United States; University Program in Genetics and Genomics, Duke University School of Medicine, Durham, NC 27710, United States; Department of Molecular Genetics and Microbiology, Duke University School of Medicine, Durham, NC 27710, United States; Department of Molecular Genetics and Microbiology, Duke University School of Medicine, Durham, NC 27710, United States; Department of Molecular Genetics and Microbiology, Duke University School of Medicine, Durham, NC 27710, United States; University Program in Genetics and Genomics, Duke University School of Medicine, Durham, NC 27710, United States; Department of Molecular Genetics and Microbiology, Duke University School of Medicine, Durham, NC 27710, United States; University Program in Genetics and Genomics, Duke University School of Medicine, Durham, NC 27710, United States; Department of Molecular Genetics and Microbiology, Duke University School of Medicine, Durham, NC 27710, United States; Department of Molecular Genetics and Microbiology, Duke University School of Medicine, Durham, NC 27710, United States; University Program in Genetics and Genomics, Duke University School of Medicine, Durham, NC 27710, United States

## Abstract

**Summary:**

Many existing software libraries for genomics require researchers to pick between competing considerations: the performance of compiled languages and the accessibility of interpreted languages. Go, a modern compiled language, provides an opportunity to address this conflict. We introduce Gonomics, an open-source collection of command line programs and bioinformatic libraries implemented in Go that unites readability and performance for genomic analyses. Gonomics contains packages to read, write, and manipulate a wide array of file formats (e.g. FASTA, FASTQ, BED, BEDPE, SAM, BAM, and VCF), and can convert and interface between these formats. Furthermore, our modular library structure provides a flexible platform for researchers developing their own software tools to address specific questions. These commands can be combined and incorporated into complex pipelines to meet the growing need for high-performance bioinformatic resources.

**Availability and implementation:**

Gonomics is implemented in the Go programming language. Source code, installation instructions, and documentation are freely available at https://github.com/vertgenlab/gonomics.

## Graphical abstract

**Figure:**
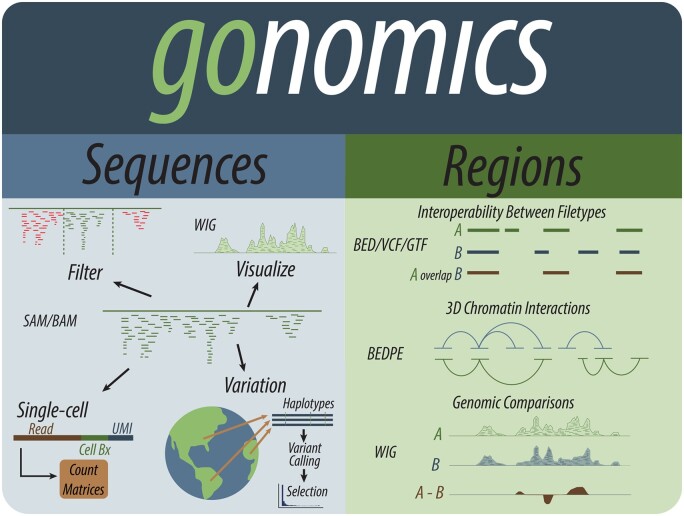

## 1 Introduction

Genomics draws the interdisciplinary expertise of biologists, computer scientists, and statisticians into its melting pot. This core strength of genomics would be best supported by a programming language that is both widely accessible and performs efficiently.

Both high-level and low-level languages have driven genomics research. High-level interpreted languages are popular because programs can be written with relatively few lines of code and are often more accessible to those with less computer science experience (Cock *et al.* 2009, McLaren *et al.* 2016, Butler *et al.* 2018, Mousavi *et al.* 2021, Abraham *et al.* 2023). Low-level compiled languages offer increased runtime efficiency, which is crucial as genomic data sets grow in size and complexity (Quinlan and Hall 2010, Kent *et al.* 2010, Hickey *et al.* 2020, Bonfield *et al.* 2021). The Go programming language (https://go.dev) is well-suited for collaborative genomics research because it unites advantages of high-level languages, such as readability and a large standard library, with advantages of low-level languages, such as compilation, static typing, and near-C runtime efficiency (Li *et al.* 2009, Neph *et al.* 2012, Shen *et al.* 2016, Kortschak *et al.* 2017, Costanza *et al.* 2019a,b).

Here we present Gonomics, a free and open-source collection of both command line tools and software libraries implemented in the Go programming language.

## 2 Features and methods

### 2.1 Gonomics at a glance

Gonomics is designed to benefit both software users and developers. For software users, we have a variety of general-purpose command line tools to accomplish common bioinformatic tasks. For developers, our library structure enables the rapid development of custom tools by offering many foundational functions, such as: reading and writing popular file formats, sorting, subsetting, intersecting, simulating, and calculating statistics. Most genomics researchers reside on the continuum between only running executables and only developing new software. Therefore, we prebuilt tools that can be easily integrated into pipelines containing both custom and existing programs in an efficient manner.

We implemented Gonomics with an emphasis on performance, rigor, and reproducibility. Gonomics programs have runtimes and memory footprints that are largely similar to popular software packages that use lower-level languages, such as C and C++ (Supplementary Data). We have chosen human-readable program, function, and variable names so that users and developers can intuitively understand our software. The Go standard library documentation, as well as all documentation for Gonomics, is automatically generated, maintained, and hosted through a web interface at https://pkg.go.dev/github.com/vertgenlab/gonomics. Additionally, we have implemented a robust testing framework to ensure stability and correctness throughout the codebase. Some commands within Gonomics have already been used in research projects (Ren *et al.* 2021, Snellings *et al.* 2022, Saelens *et al.* 2022, Mangan *et al.* 2022), but by releasing the entire repository and using continuous integration, we hope to increase engagement with the genomics community and ensure a robust codebase with expanding functionality.

### 2.2 One file operations

Gonomics can handle and manipulate data from a wide range of standard bioinformatic file formats including sequence formats (e.g. FASTA and FASTQ), alignment formats (e.g. SAM, BAM, MAF, AXT, and CHAIN), and annotation formats (e.g. WIG, BED, BEDPE, VCF, GENEPRED, and GTF). Gonomics offers an array of existing command line tools for operations on these file formats including filtering (e.g. *bedFilter*, *vcfFilter*, and *faFilter*), formatting (e.g. *bedFormat*, *vcfFormat*, and *faFormat*), and sorting (e.g. *mergesort*). Other tools simulate random datasets (e.g. *randSeq*, *simulateSam*, and *simulateVcf*) or perform mathematical operations (e.g. *bedDistanceFromChrEnds* and *wigMath*) (Table 1). For memory efficiency and concurrency, we extensively use channels to stream data, which are central features of the Go standard library.

**Table 1.** Examples of command line tools in Gonomics.

Category	Command	Description
Variation	callVariants	Call somatic variants from aligned reads
	vcfFilter	Filter the variants present in a VCF file based on their frequency in the population, type of mutation, alleles in specific individuals, or any value associated with the INFO field
	vcfAfs	Calculate the allele frequency spectrum from a VCF file
	haplotypeGenerator	Generate all possible haplotypes observed in a VCF file over a genomic interval
Single-cell	fastqFormat	Parse UMIs and cell barcodes from reads in a FASTQ file
	scCount	Create a count matrix based on a SAM/BAM file and a GTF file, allows input-normalization
Simulation	simulateSam	Simulate aligned Illumina-style reads from a FASTA file
	simulateVcf	Create a VCF file of variants and their allele frequencies based on a selection parameter acting on those variants
Visualization	multiFaToVcf	Create a VCF file based on a multiple alignment in FASTA format
	samToWig	Convert a SAM/BAM file into a WIG file for visualizing reads
	wigMath	Allow smoothing or basic mathematical transformations of values in a single WIG file, or element-wise mathematical operations when given two WIG files
3D contacts	bedpeOverlap	Filter elements of a BEDPE file based on whether one or both feet overlap entries in another BED or BEDPE file
	intervalContacts	Filter a file of genomic intervals (e.g. BED, VCF, or SAM/BAM) based on whether each entry overlaps either foot of a BEDPE file
Interoperability	mergesort	Sort entries in a file based on genomic coordinates (e.g. BED, VCF, or SAM/BAM) or single-cell barcodes
	intervalOverlap	Detect overlapping genomic regions across multiple file types (e.g. BED, VCF, or SAM/BAM)
	overlapEnrichments	Calculate the statistical significance of enrichment/depletion of elements in one file that overlap elements in a second file
	liftCoordinates	Update the coordinates for a variety of file types (e.g. BED, VCF, or SAM/BAM) based on an alignment between reference genomes

We propose that genomics research will increasingly incorporate single-cell technologies, population genetics, and 3D chromatin structure. Therefore, we have implemented tools to parse cell barcodes, filter unique molecular identifier duplicates, and generate count matrices from 10x-format single-cell sequencing data. For population genetics, we have built tools related to somatic variant calling (*callVariants*), allele frequency spectra (*vcfAfs*), and selection analysis (*selectionMcmc*). Gonomics also provides a package for reading, writing, and intersecting BEDPE files, which encode interactions between distant genomic regions in 3D space (Table 1).

### 2.3 Interfacing between filetypes

While Gonomics has many tools for filetype conversions, it is often preferable to interface between file formats. Conversions create additional intermediate files, computations, and input/output operations (I/O), reducing performance for large datasets. Furthermore, data fields in source file formats will be lost in conversions if they are not represented in destination formats. An example of interoperability in our repository is the *Interval* interface, which allows us to encode data on genomic intervals across many common bioinformatic file formats, including BED, VCF, and SAM/BAM. Powered by the *Interval* interface and range trees (Mao *et al.* 2019), the program *intervalOverlap* allows the user to identify all overlapping genomic intervals between two files, such as all variants from a VCF file overlapping all regions in a BED file, without the need to convert. The program *overlapEnrichments* also uses the *Interval* interface to calculate statistical enrichments and depletions for overlaps between elements. As new reference genomes become available, existing data sets need to be ‘lifted’ to new coordinate systems and *liftCoordinates* accomplishes this conversion for all filetypes that satisfy the *Interval* interface (Table 1). Thus, interoperability enables Gonomics to integrate efficiently across diverse file types.

### 2.4 Modular tool development

While Gonomics contains many prebuilt general-purpose command line programs, its modular library structure enables the rapid development of more specific tools. Below we present an example code block that reads in a DNA sequence from a file in FASTA format and prints the translated amino acid sequence to the user. We utilize our *fasta* and *dna* packages as building blocks to condense the required code to 8 lines. While this same task could be implemented in a slightly more condensed format, we prioritize explicit variable declarations with readable names throughout Gonomics.

*// Gonomics autodetects the .gz extension*

**var**seqs []dna.Base = fasta. Read(“dna.fa.gz”)

**var**prot []dna.AminoAcid

**var**prettyProt**string**

**for**i :=**range**seqs {

  prot = dna. TranslateSeq(seqs[i].Seq)

  prettyProt = dna. PeptideToString(prot)

  fmt.Print(seqs[i].Name, prettyProt)

}

Gonomics libraries can also be used in a complementary fashion with other bioinformatics projects written in Go. Bíogo (Kortschak *et al.* 2017) is a set of high-quality bioinformatic libraries written in Go, which offer functionality not present in Gonomics, such as support for CRAM and TABIX file types. The performance of shared functionality between Gonomics and bìogo is largely similar, with runtime and memory usage usually being within a factor of two of each other (Supplementary Table S1). However, the coding style can be quite different between the two projects with Gonomics having a more procedural style and bíogo having a more object-oriented style. There will also be times when a programmer needs functionality that is not present in an existing Go library; however, projects such as elPrep (Costanza *et al.* 2019a,b) have code to interface between the Go programming language and many popular genomics software tools in a time-efficient manner. Gonomics, in combination with these other projects, offers a solid foundation for future genomics software projects written in Go.

## 3 Conclusion

Gonomics is an open-source collection of command line programs and bioinformatics libraries in the Go programming language that unites the readability of high-level interpreted languages with the performance of low-level compiled languages. Gonomics can read, write, and manipulate data in the major bioinformatic file formats as well as convert and interface between these formats. The modular structure of Gonomics libraries enables the development of high-performance programs using human-accessible code. As genomic analyses grow in both their computational demands and biological complexity, we anticipate that platforms like Gonomics will become increasingly important to foster effective interdisciplinary collaborations.

## Supplementary Material

btad516_Supplementary_Data

## Data Availability

The Gonomics source code (v1.0.0) is available at: https://github.com/vertgenlab/gonomics. Software and data sets used during benchmarking are available at: https://github.com/vertgenlab/benchmarks.
