# Supplementary material for: Gonomics: uniting high performance and readability for genomics with Go
Source: Bioinformatics. 2023 Aug 25;39(8):btad516. doi: 10.1093/bioinformatics/btad516 (PMC10466080; doi:10.1093/bioinformatics/btad516)
Supplement: btad516_Supplementary_Data [file btad516_supplementary_data.pdf]

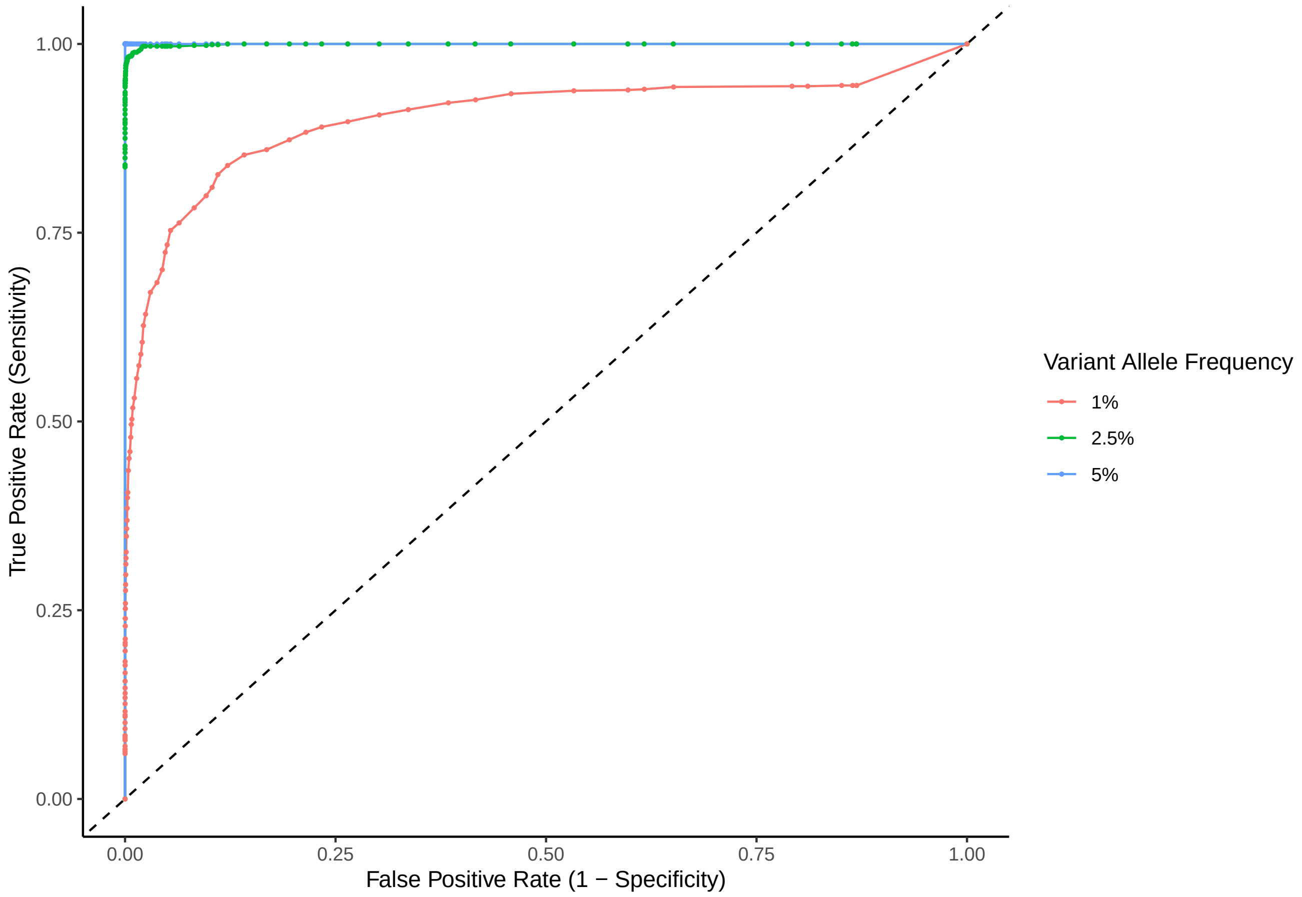

Supplementary Figure S1: **Performance of *callVariants* on simulated data.** Reads were generated against a 1mb fasta file to 1000x coverage with a per-base quality of 20 (99% sequencing accuracy) using *simulateSam -coverage 1000 -flatErrorRate 0.01*. Variants were introduced at varying allele frequencies by randomly subsetting simulated BAM files containing 100% reference alleles and 100% alternate alleles. Variants were called from bam files using permissive filters *callVariants -p 2 -r 1mb.fa -minAF 0 -maxStrandBias 1*; the normal for these runs was a 1000x BAM file with 100% reference alleles generated identically as above with a different seed. Shown here are the sensitivity and specificity values of the caller at various p-value thresholds for various allele frequencies. The curves for all frequencies >5% are identical to the 5% curve and are thus omitted here. The code and raw data used to generate this plot are available at https://github.com/vertgenlab/benchmarks.

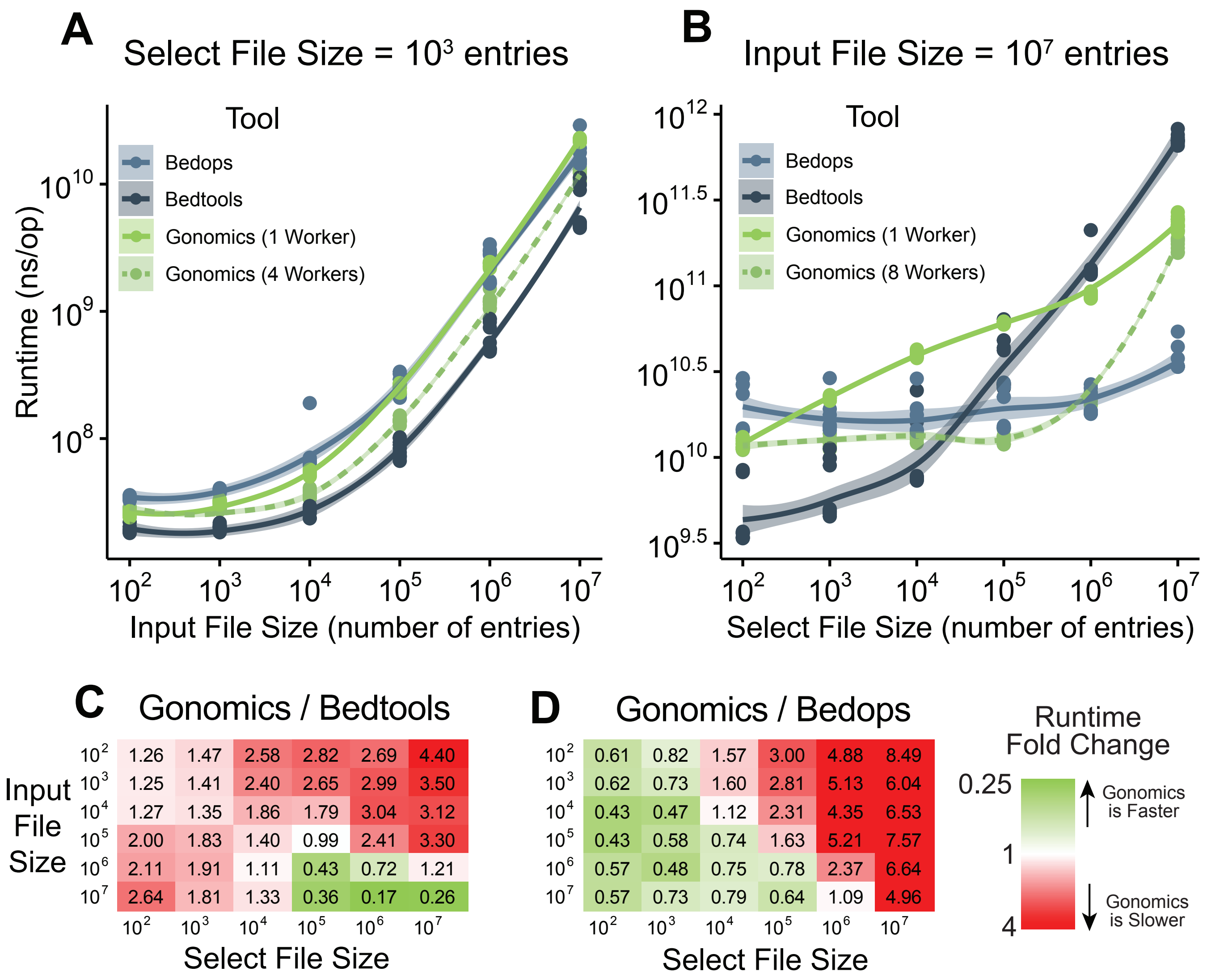

Supplementary Figure S2: **Comparative benchmarking for *gonomics intervalOverlap* (with either 1, 4, or 8 workers), *bedtools intersect*, and *bedops –element-of*.** All three programs return the set of all entries in an input file that overlap entries in a select file. Runtime in units of nanoseconds per operation is displayed for either increasing input file size and fixed select file size (**A**) or increasing select file size and fixed input file size (**B**). Unsorted input and select files in BED format were simulated as random regions in the human reference genome *hg38*. *bedtools intersect* automatically detects that the file is unsorted and uses more runtime than if it were given a sorted file. As *bedops* only accepts sorted BED files, we call *bedops sort-bed* with default options as part of the *bedops* benchmark. Ten replicate benchmarks were run for each file size combination. (**C**, **D**) Heatmaps display the runtime fold change comparing *gonomics intervalOverlap* (8 workers) to either *bedtools intersect* (**C**) or *bedops –element-of* (**D**). Runtime fold change is estimated as the average across ten replicate benchmarks. The code and raw data used to generate these plots are available at https://github.com/vertgenlab/benchmarks.

Supplementary Table S1: File I/O performance of gonomics and biogo.

File Type	Operation	gonomics Runtime(ms)	biogo Runtime(ms)	gonomics Memory(MB)	biogo Memory(MB)
BAM (10^4 records)	Read	38	33	3.8	10.4
	Write	126	60	6.2	3.1
BED (10^4 records)	Read	1.50	1.56	1.0	1.6
	Write	2.70	3.02	1.6	0.49
FASTA (10^6 bases)	Read	47	35	78	83
	Write	21	94	29	10
FASTQ (10^4 records)	Read	19	17	17	20
	Write	12	59	11	6.2
GTF ($4 * 10^4$ transcripts)	Read	886	1433	685	1229
	Write	686	997	495	280
SAM (10^4 records)	Read	22	15	15	22
	Write	14	22	16	20

Benchmarks were performed using the builtin Go benchmarking utility on a machine with 4x 3.2GHz and 4x 2.064 GHz arm64 CPUs, and 16GB of RAM running macOS12.1. Reported values are an average from 200x iterations. All functions with a threads argument were run with a single thread. The code used to generate these benchmarks is available at https://github.com/vertgenlab/benchmarks.

Supplementary Table S2: Bam reading benchmarks against commonly used tools.

Program	Runtime (s)	Memory (MB)
gonomics	3.70	11.3
biogo	3.14	11.3
samtools	1.62	3.80
pysam[1]	1.75	16.8
elprep[1]	3.10	1957[2]

All values are for reading a sorted, indexed bam file containing 10^6 records. Benchmarks were performed using the builtin Go benchmarking utility on a machine with 4x 3.2GHz and 4x 2.064 GHz arm64 CPUs, and 16GB of RAM running macOS12.1. Each benchmark was performed by calling the binary for the respective program with the exec package in Go. Reported values are an average from 10x iterations. All functions with a threads argument were run with a single thread. The code used to generate these benchmarks is available at https://github.com/vertgenlab/benchmarks.

[1]Both pysam and elprep interface with samtools/htslib for reading bam files.

[2]elprep is designed to store data in RAM during computation accounting for the large memory footprint.

Supplementary Table S3: Comparative benchmarking for gonomics intervalOverlap against bedtools and bedops.

Select File Size (number of entries)	10^2	10^2	10^7	10^7
Input File Size (number of entries)	10^2	10^7	10^2	10^7
Program	Memory (KB)	Memory (KB)	Memory (KB)	Memory (KB)
gonomics (1 worker)	13268	19244	13271332	15039484
gonomics (8 workers)	13440	18120	13052600	14986164
bedtools	3848	4152	3362556	3362568
bedops (sort select file)	1896	1952	247224	250160
bedops (sort input file)	1948	249416	1944	248692
bedops (sorted input)	16196	16356	16356	16356

Benchmarks were conducted with *gonomics intervalOverlap* (with either 1 or 8 workers), *bedtools intersect*, and *bedops –element-of*. All three programs return the set of all entries in an input file that overlap entries in a select file. Unsorted input and select files in BED format were simulated as random regions in the human reference genome *hg38*. As *bedops* only accepts sorted BED files, we call *bedops sort-bed* with default options as part of the *bedops* benchmark. The code used to generate these benchmarks is available at https://github.com/vertgenlab/benchmarks.
